# Clinical study of separation surgery combined with intraoperative vertebroplasty and ^125^I seeds implantation for spinal metastases from lung adenocarcinoma with epidural spinal cord compression

**DOI:** 10.1186/s12957-025-04148-8

**Published:** 2025-12-12

**Authors:** Ya Zhang, Bin Fan, Tiying Wang, Yihao Yang, Nabil Zia UI Haq, Zhou Huang, Dachang Xu, Yan Liu, Dongqi Li, Lei Han, Weiquan Wang, Linhao Cai, Xiaohui Yang, Hongpu Sun, Jing Zhang, Cao Wang, Tao Yuan, Xiang Ma, Zuozhang Yang

**Affiliations:** Bone and Soft Tissue Tumors Research Center of Yunnan Province, Department of Orthopaedics, The Third Affiliated Hospital of Kunming Medical University, Yunnan Cancer Hospital, Peking University Cancer Hospital Yunnan, Kunming, Yunnan 650118 China

**Keywords:** Separation surgery, vertebroplasty combined with interstitial implantation ^125^I of seeds(VPI), Spinal metastases, Lung adenocarcinoma, Metastatic Epidural spinal cord compression

## Abstract

**Background:**

The study aim was to evaluate the efficacy and safety of separation surgery combined with intraoperative vertebroplasty and^125^I seeds implantation for the treatment of lung adenocarcinoma-associated metastatic epidural spinal cord compression (MESCC).

**Methods:**

We retrospectively analyzed patients with lung adenocarcinoma spinal metastases who were treated for epidural spinal cord compression. Patients were divided into the SSVPI group (Separation surgery + vertebroplasty + ^125^I seeds implantation, n=48) and the control group (Decompression surgery involving separation, reconstruction, and fixation, n=48). At postoperative follow-up (1 week to 12 months), spinal cord function, pain, performance status, muscle strength, and health-related quality of life were assessed. Complications were recorded, and overall survival, progression-free survival, and local control were analyzed using Kaplan-Meier curves.

**Results:**

All 96 patients underwent the surgery successfully. The baseline characteristics showed no significant between the two groups. The SSVPI group demonstrated superior outcomes, with better neurological recovery(Higher International Standards for Neurological Classification of Spinal Cord Injury grade D improvement; >40% at 1/3/6 months, P<0.005). Pain relief (Faster visual analog scale pain score reduction;>60% decline within 2 months, P<0.001), and improved performance status (Earlier ECOG/KPS improvement;≥75% by 2 weeks). Quality of life, assessed by Quality of Life Questionnaire-Core 30 scores increased by 18.6 points at 3 months (P≤0.003).The SSVPI group also experienced longer median overall survival (20 vs. 12 months, P=0.026), progression-free survival (12 vs. 8 months), and local control (all P<0.05). The SSVPI group primarily experienced minor bone cement leakage (10.42%)), whereas the control group experienced complications predominantly related to tumor progression-associated secondary spinal cord compression (3 cases, 6.25%) and radiation therapy-related adverse events (total incidence: 39.6%), including radiation dermatitis (18.75%), osteonecrosis (16.67%), and spinal cord injury (4.17%).

**Conclusion:**

SSVPI is an optimized treatment for lung adenocarcinoma-related MESCC. It provides immediate spinal cord decompression and stabilization, concurrent intraoperative ^125^I seeds implantation for early and sustained radiotherapy, and avoids delayed postoperative radiation and complications associated with external beam therapy. This approach enhances local tumor control while preserving neurological function and improving quality of life.

## Introduction

With the development of treatment strategies for advanced lung adenocarcinoma, widespread adoption of multidisciplinary team approaches, advancements in surgical techniques, and the extensive application of targeted therapy and immunotherapy [1–3], patient survival has significantly improved, leading to an increased incidence of bone metastases. The spine is the most common site of osseous metastasis in malignant tumors [4], is frequently involved, resulting in skeletal-related events such as intractable back pain, pathological fractures, and hypercalcemia. The aggressive growth of metastatic lesions can lead to osteolytic destruction of vertebral bodies and their appendages, subsequently causing vertebral compression fractures and spinal instability. Notably, 5%−20% of patients develop metastatic epidural spinal cord compression (MESCC) [5, 6] ^,^ with approximately 5–10% [7, 8]experiencing irreversible paralysis due to spinal cord compression, severely compromising their quality of life.

Surgical intervention remains a cornerstone in the comprehensive management of spinal metastases.Although total en bloc spondylectomy (TES) enables radical tumor resection, its clinical application is limited due to significant intraoperative blood loss, technical complexity, and prolonged recovery [9]. Radiation therapy, though effective in pain relief, suffers from delayed onset and inability to restore spinal stability, potentially exacerbating pathological fractures [10]. In 2010, Bilsky’s team [11] introduced an innovative paradigm of separation surgery (SS) combined with stereotactic body radiotherapy (SBRT). This approach achieves 360° spinal cord decompression with a 2–3 mm tumor-dura safety margin while preserving vertebral structure. Compared to TES, this protocol demonstrates significantly reduced surgical trauma (50% shorter operative time, 60% less blood loss) [7, 12]. Clinical data from Laufer et al. [13] confirmed a median progression-free survival of 14.5 months with this combined modality. However, three major challenges persist: Spinal instability, Radiation-related complications (8–12% osteonecrosis, 15–20% grade ≥ III dermatitis) [14–16], Limited tumor control (18–22% in-field recurrence, 30% epidural recurrence) [15].

Recent advances in percutaneous vertebroplasty with ^125^I seeds implantation (PVPI) [17, 18] have demonstrated unique advantages for palliative treatment of multilevel metastases, including minimally invasive access, rapid analgesia, and low complication rates. Building on this, we developed an innovative SSVPI technique(integrating SS and VPI)to treat MESCC in patients with lung adenocarcinoma. Preliminary clinical observations demonstrate its significant therapeutic advantages.

This study aims to comprehensively evaluate the safety, feasibility, and clinical efficacy of SSVPI in patients with MESCC, thereby providing novel evidence-based insights for the multidisciplinary management of spinal metastases in lung adenocarcinoma.

## Methods

### Study design and study population

This retrospective study analyzed clinical data from 96 patients diagnosed with lung adenocarcinoma spinal metastases complicated by MESCC and treated at the Department of Orthopedics, the Third Affiliated Hospital of Kunming Medical University, between July 2018 and March 2025. Surgical intervention was performed in all patients, with adjunctive treatments administered in strict accordance with National Comprehensive Cancer Network (NCCN) guidelines for spinal metastases management. Patients were divided into two groups according to the treatment they received: the SSVPI group (Separation surgery with vertebroplasty and ^125^I seeds implantation, *n* = 48) and the Control group (Decompression surgery involving separation, reconstruction, and fixation, *n* = 48).

### Clinical data collection

Comprehensive baseline data were collected for both groups, including: Age, Sex, Number of vertebral metastases, Involved spinal levels, Driver gene mutation status, Spinal Instability Neoplastic Score (SINS), Duration of neurological deficits, intraoperative blood loss, Operative time, International Standards for Neurological Classification of Spinal Cord Injury (ISNCSCI) impairment scale, ECOG performance status, Visual Analog Scale (VAS) for pain, Karnofsky Performance Status (KPS), Walking Ability Score (WAS), Lovett muscle strength grading, European Organization for Research and Treatment of Cancer Quality of Life Questionnaire-Core 30 (EORTC QLQ-C30) physical function scores, Follow-up duration. All parameters were systematically recorded for comparative analysis between groups.

### Inclusion and exclusion criteria

Inclusion Criteria were Confirmed diagnosis of lung adenocarcinoma with spinal metastases and MESCC through clinical, imaging, and pathological examinations; life expectancy ≥ 3 months as assessed by the modified Tokuhashi score; neurological impairment with ISNCSCI grades A-D; Epidural Spinal Cord Compression (ESCC) score ≥ 1; spinal Instability Neoplastic Score (SINS) > 7 (indicating spinal mechanical instability); absence of severe cardiac, pulmonary, or cerebral comorbidities that would contraindicate surgery; written informed consent obtained from both patients and their families; systemic therapy administered in strict accordance with NCCN guidelines; compliance with scheduled hospital follow-ups and telephone surveillance post-treatment.

Exclusion Criteria were patients with spinal metastases without incomplete or complete paraplegia; cases without epidural spinal cord compression (ESCC grade 0); spinal Instability neoplastic Score (SINS) < 7; life expectancy < 3 months as assessed by the modified Tokuhashi score; presence of absolute surgical contraindications precluding operative tolerance; prior history of spinal surgery for metastases or other spinal pathologies; patients or family members declining surgical intervention; failure to complete planned comprehensive treatment (pre- or post-operatively); and inability or refusal to participate in clinical data collection or follow-up due to objective reasons.

### Treatment

#### SSVPI group: separation surgery combined with intraoperative vertebroplasty and ^125^I seeds implantation

**Fig. 1:**
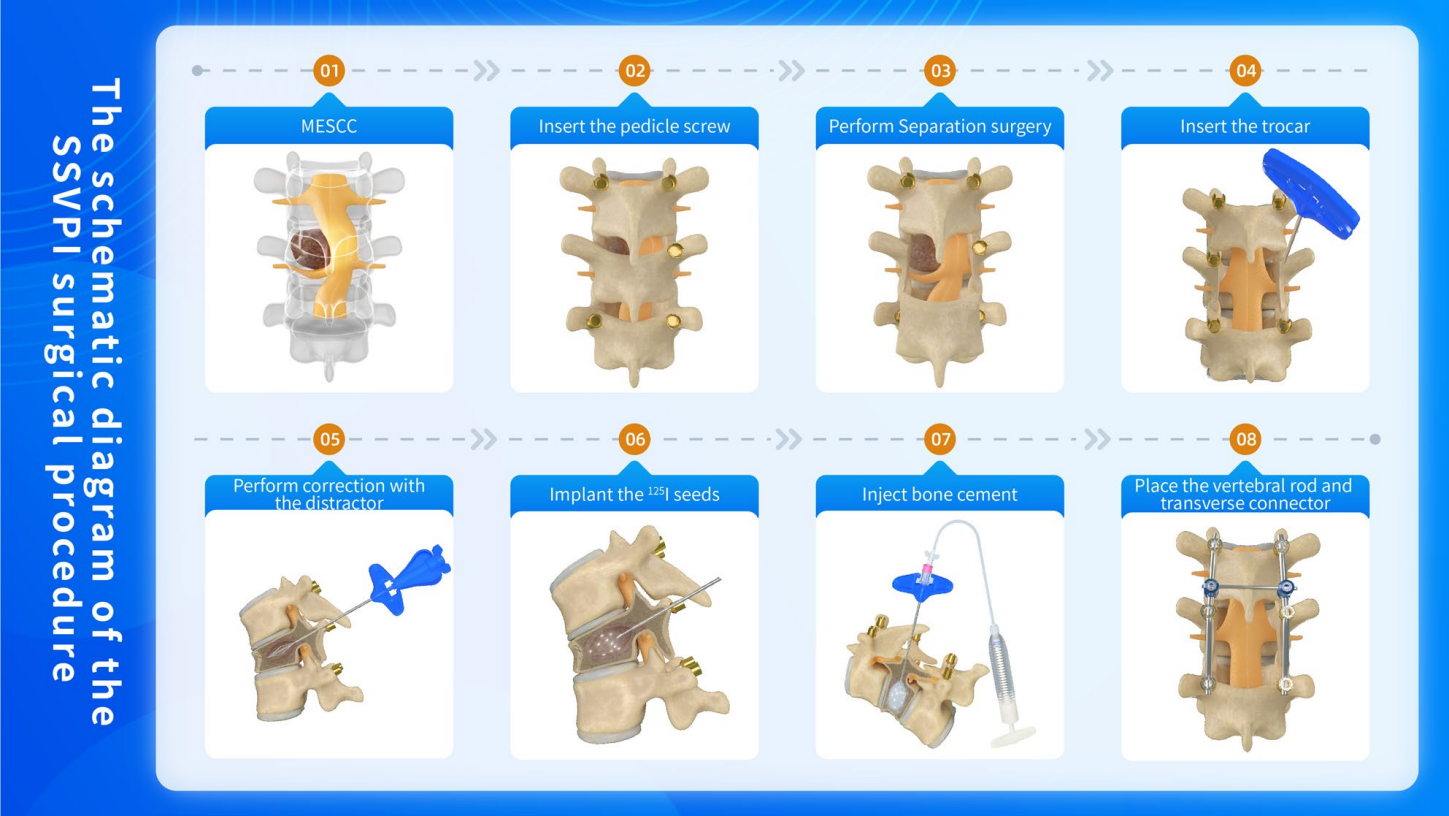
The schematic diagram of the SSVPI surgical procedure

After successful induction of anesthesia, the patient was placed in the prone position. The procedure was performed as follows: Separation Surgery: The initial separation procedure was performed to achieve neural decompression while maximizing preservation of the vertebral bony architecture. Vertebral Body Access: Under direct visualization, the affected vertebral body was punctured. Lateral fluoroscopy confirmed proper needle placement at the junction of the anterior and middle thirds of the vertebral body. The needle tip was advanced to the central vertebral region before stylet removal.Vertebral Venography: Contrast medium was injected to visualize vascular anatomy. Residual contrast and blood were aspirated to reduce intralesional pressure. ^125^I Seeds Implantation: Preoperative Treatment Planning System (TPS) calculations determined the required number of ^125^I seeds. Seeds were implanted through the access needle into the target area. Multiple needle placements or trajectory adjustments were made as needed to achieve optimal three-dimensional seed distribution based on tumor characteristics. PMMA Cement Augmentation: Intravenous dexamethasone (10 mg) was administered prophylactically. Polymethylmethacrylate (PMMA) cement (Tianjin Synthetic Material Research Institute, China) was mixed with contrast to optimal viscosity and injected in the “toothpaste phase” under low pressure. Continuous needle rotation during injection ensured uniform cement distribution. Direct visualization and fluoroscopic monitoring prevented posterior cement leakage. Any extravasated cement was immediately removed. Cement volume: 3.8–6.3 mL (mean 4.95 mL). Instrumentation and Closure:The stylet was reinserted and the needle withdrawn with rotation. Titanium rods were contoured to match spinal curvature and secured to pedicle screws.The surgical site was irrigated, hemostasis achieved, and a closed suction drain placed.Wound closure was performed in layers. Refer to Fig. 1 for the surgical workflow schematic.

#### Control group: decompression surgery involving separation, reconstruction, and fixation

After successful anesthesia, the patient was placed in the prone position. Intraoperative C-arm fluoroscopy was used to determine the target vertebral level. Surgical Exposure: A midline posterior incision was made to expose the affected vertebra and 2–3 adjacent levels above and below. The spinous process, lamina, and superior/inferior articular processes were fully visualized. Posterior Instrumentation & Decompression: Pedicle screws of appropriate length were placed under fluoroscopic guidance for posterior stabilization. The spinous process, lamina, posterior longitudinal ligament, and compressive lesions were resected to achieve complete spinal canal decompression. Following measurement of the vertebral defect, a titanium cage filled with bone cement was placed to achieve structural reconstruction, No en bloc or piecemeal tumor resection was performed. Neurological protection: Segmental nerves were preserved where possible. Bilateral T2 and lower intercostal nerves were ligated/resected if tumor removal required. Final Fixation & Closure: Titanium rods were contoured to match spinal curvature and secured to pedicle screws.The surgical site was irrigated, hemostasis achieved, and a closed suction drain placed. Layered wound closure was performed. Subsequent adjuvant therapies were strictly administered in accordance with the NCCN Guidelines for Management of Metastatic Spinal Tumors.

#### Postoperative management

All patients received prophylactic antibiotic therapy for 1–2 weeks postoperatively. Motor and sensory functions of the lower extremities were closely monitored. The patients were instructed to maintain regular axial turning and receive back percussion to prevent pressure sores and hypostatic pneumonia. Early postoperative lower extremity functional exercises were performed to prevent deep vein thrombosis (DVT). Postoperative digital radiography (DR) was performed to assess the position of the internal fixation devices and detect any loosening or breakage. For patients who underwent SSVPI, additional DR and Computed tomography(CT) scans were performed to evaluate the distribution of bone cement and radioactive particles. Negative pressure drainage devices were removed when the 72-hour drainage volume was less than 30 mL in both patient groups. All patients underwent surgical treatment, with additional therapies administered in accordance with the NCCN Clinical Practice Guidelines for the management of spinal metastases from lung adenocarcinoma.

#### Radiation protection protocol for ^125^I seeds implantation

Given the continuous low-dose-rate emission (half-life = 59.4 days) of ^125^I seeds, our comprehensive radiation protection protocol included: (1) intraoperative shielding (lead aprons, eyewear, and ALARA (As Low As Reasonably Achievable) principle, with additional lead drapes for SSVPI cases); (2) postoperative management (activity restrictions, > 1 m distancing from vulnerable groups for 1 week); (3) safe seed disposal in lead containers; and (4) patient/public education on risk mitigation. The SSVPI group patients received enhanced measures, including surgical-site lead shielding and structured safety instructions. This multi-level approach effectively minimized radiation exposure while ensuring therapeutic efficacy.

#### Observation indicators and follow-up

Patients in both groups were followed up at 1 week, 2 weeks, 1 month, 2 months, 3 months, 6 months, and 12 months postoperatively. The following assessment tools were used to compare changes in spinal cord function, physical performance, pain scores, and health-related quality of life between the two groups before and after surgery: ISNCSCI grading, ECOG score, VAS score, KPS score, WAS score, Lovett muscle strength grading, and EORTC QLQ-C30 physical function score. Complications were also observed and recorded.

Additionally, follow-up indicators including overall survival (OS), progression-free survival (PFS), and local recurrence rate were monitored. Survival curves were plotted and survival analysis was performed. Based on clinical symptoms, physical signs, and imaging data, the presence of severe complications was determined, including radiation-induced spinal cord injury, radiation dermatitis, osteoradionecrosis, cerebrospinal fluid leakage, bone cement leakage, internal fixation loosening or breakage, and pulmonary embolism.

#### Statistical methods

Statistical analysis was performed using SPSS 25.0 software. Continuous variables were tested for normality using the Shapiro-Wilk test. Normally distributed data were analyzed using paired-sample t-tests, while non-normally distributed or ordinal data were analyzed using the Mann-Whitney U test. Survival time was defined as the duration from the diagnosis of spinal metastasis from lung adenocarcinoma to local recurrence, disease progression, or death. Kaplan-Meier analysis was used to generate survival curves. P-value < 0.05 was considered statistically significant.

## Results

### General characteristics

**Fig. 2:**
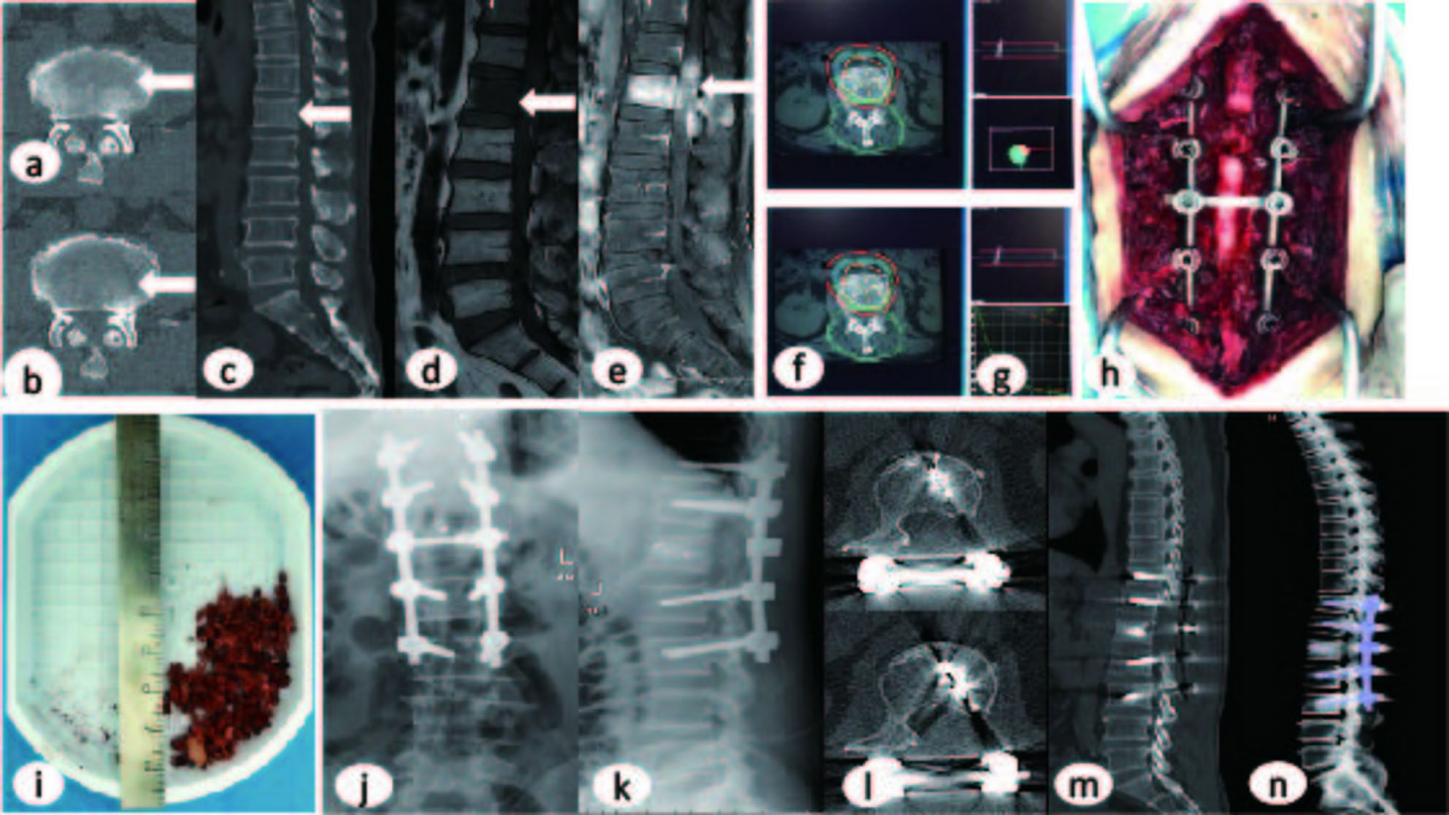
Preoperative Imaging (**a**-**e**): CT/MRI demonstrated osteolytic destruction of L1 vertebral body and posterior elements with epidural tumor extension. Treatment Planning (**f**-**g**): Target volume calculation using Treatment Planning System (TPS) determined the required number of ^125^I seeds. Intraoperative Procedure (**h**): Successful L1 SSVPI (Separation Surgery Combined with Intraoperative Vertebroplasty and^125^I Seeds Implantation) performed; (**i**): the postoperative pathological specimen Postoperative Imaging (**j**-**n**): DR/CT confirmed:Proper positioning of pedicle screw-rod system at T11/T12/L2/L3 levels. No evidence of implant migration, loosening, or fracture. Multiple linear metallic seed trajectories and patchy high-density cement shadows within L1 vertebra Current Status:The patient remains clinically stable with ongoing follow-up monitoring

**Fig. 3:**
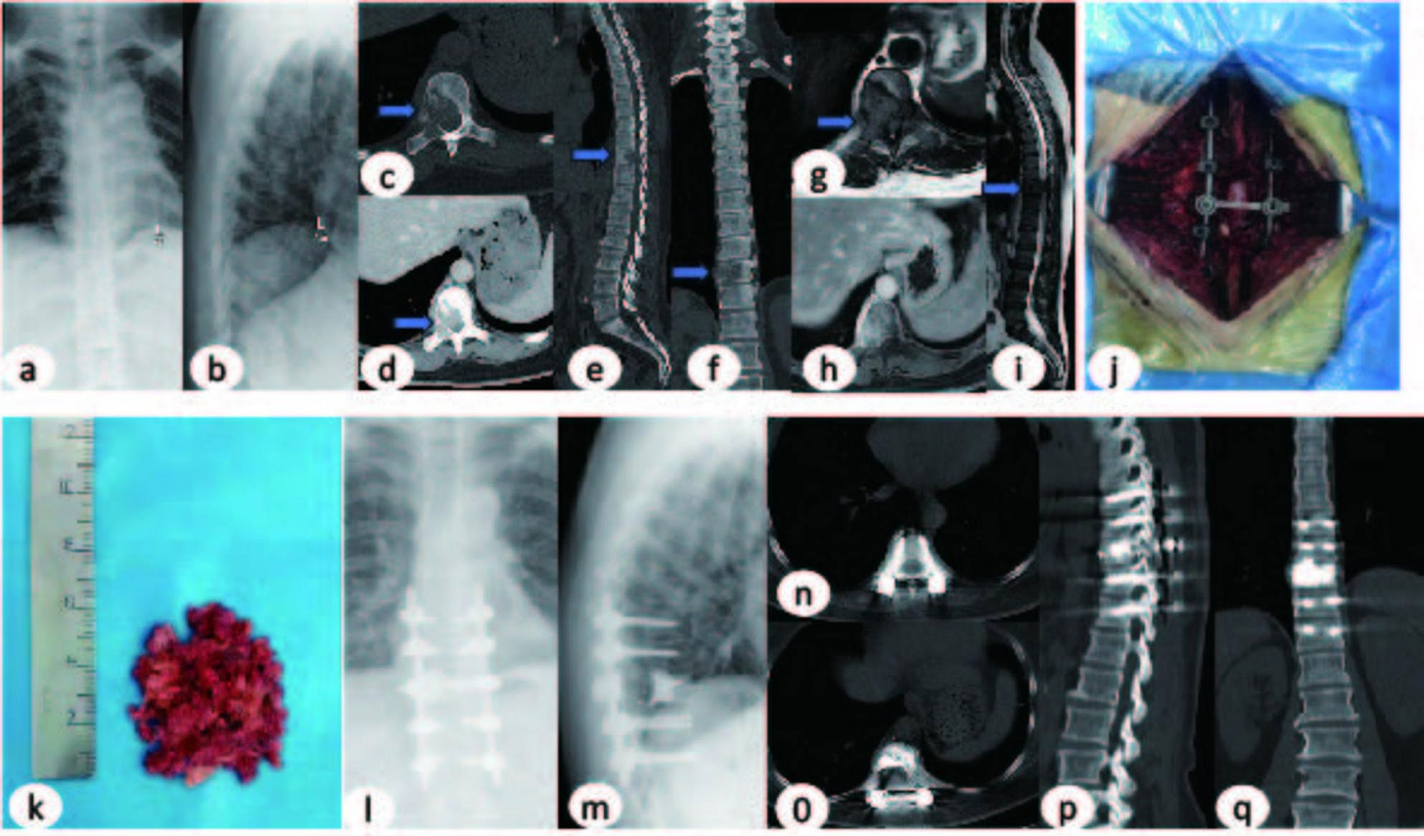
Preoperative Imaging (**a**-**i**): X-ray/CT/MRI revealed osteolytic destruction of the left T10 vertebral body with associated paravertebral soft tissue mass, demonstrating significant epidural extension causing spinal canal compromise. Intraoperative Procedure (**j**): Successful T10 SSVPI (Separation Surgery Combined with Intraoperative Vertebroplasty and 125I Seeds Implantation) performed; (**k**): the postoperative pathological specimen Postoperative Imaging (**l**-**q**): DR/CT confirmed:Proper positioning of pedicle screw-rod system at T8/T9/T11/T12 levels. No evidence of implant migration, loosening, or fracture. Multiple linear metallic seed trajectories and patchy high-density cement shadows within T10 vertebra Current Status: The patient remains clinically stable with ongoing follow-up monitoring

This study comprised 45 male and 51 female patients, aged 26–79 years (mean 57.375 ± 11.09 years). All 96 patients successfully completed the surgical procedures. Complete follow-up data were obtained for all 96 patients, with follow-up durations ranging from 5.23 to 24 months (mean 16.10 months). Representative pre-and postoperative imaging data are shown in Figs. 2 and 3 respectively. No statistically significant differences were observed between the study and control groups for baseline characteristics including age, gender, number of vertebral metastases, location of metastatic vertebrae, driver gene test results, SINS scores, duration of neurological deficits. (Table 1).

**Table 1:** Baseline data of SSVPI group and control group

Baseline data	SSVPI group (*n* = 48)	Control group (*n* = 48)	*P* value
Age	58.08 ± 10.72	56.67 ± 11.51	0.534
Gender			
Male	23	22	0.838
Female	25	26	
The number of diseased vertebral bodies per patient			
1	18	20	0.528
2	13	16	
≥ 3	17	12	
Driver Gene Test			
Positive	35	33	0.653
Negative/None	13	15	
Intra-operative blood loss(ml)	683.6458 ± 301.620	840.4583 ± 262.247	0.008
Duration of the surgery(min)	265.23 ± 47.24	245.17 ± 43.70	0.033
SINS score			
Intermediate-risk (7–12)	27	29	0.679
High-risk(≥13)	21	19	
Duration of neurological function deficit			
Less than 24 h	7	6	0.938
24–48 h	10	9	
48–72 h	14	13	
More than 72 h	17	20	
Preoperative ISNCSCI grade			
A	5	4	0.912
B	9	7	
C	13	15	
D	21	22	
Preoperative ECOG score	3.00(2.25,3.00)	3.00(3.00,3.00)	0.912
Preoperative VAS score	7.00(7.00,9.00)	8.00(6.25,9.00)	0.860
Preoperative KPS score	40.00(32.50,50.00)	40.00(40.00,50.00)	0.475
Preoperative WAS score	0(0,2.00)	0(0,2.00)	0.773
Preoperative Lovett grade	2.77 ± 1.31	2.19 ± 1.14	0.022
0	5(10.4)	3(6.3)	
1	9(18.8)	5(10.4)	
2	9(18.8)	14(29.2)	0.016
3	22(45.8)	4(8.3)	
4	3(6.3)	22(45.8)	
Preoperative C30 score	57.00(37.25,69.50)	55.50(43.50,68.00)	0.800

### Clinical outcomes

#### Spinal cord function

The pre-treatment ISNCSCI grade distribution showed no significant intergroup differences (*P* = 0.656). Two weeks postoperatively, both groups exhibited increased Grade D proportions (control: 79.2% vs. SSVPI: 85.4%), with no statistical significance (*P* = 0.540). One month postoperatively, the SSVPI group demonstrated a significantly higher prevalence of Grade D (91.7% vs. 66.7%, *P* = 0.003). Three months postoperatively, the SSVPI group maintained 91.7% of Grade D, whereas the control group showed 2.1% had regressed to grade A (*P* = 0.001). At 6 months, the SSVPI group sustained 91.7% grade D, whereas the control group had 62.5% grade D (*P* = 0.001). (Table 2).

**Table 2:** Neurological recovery (ISNCSCI grades) in SSVPI vs. Control groups before and after treatment [n(%)]

ISNCSCI grade	Control group	SSVPI group	Z value	*P* value
B.S				
A	4(8.3)	5(10.4)	−0.445	0.656
B	7(14.6)	9(18.8)		
C	15(31.3)	13(27.1)		
D	22(45.8)	21(43.8)		
A.S(2 weeks)				
A	0	0	−0.613	0.540
B	1(2.1)	4(8.3)		
C	9(18.8)	3(6.3)		
D	38(79.2)	41(85.4)		
A.S(1 month)				
A	0	0	−2.985	0.003
B	4(8.3)	1(2.1)		
C	12(25.0)	3(6.3)		
D	32(66.7)	44(91.7)		
A.S(3 months)				
A	1(2.1)	0	−3.211	0.001
B	5(10.4)	1(2.1)		
C	11(22.9)	3(6.3)		
D	31(64.6)	44(91.7)		
A.S(6 months)				
A	1(2.1)	0	−3.357	0.001
B	3(6.3)	1(2.1)		
C	14(29.2)	3(6.3)		
D	30(62.5)	44(91.7)		

B.S: Before surgery

A.S: After surgery

### VAS scores

No significant intergroup difference was observed in the preoperative VAS scores (*P* = 0.860), confirming balanced baseline characteristics. The SSVPI group showed superior early pain relief at 1 and 2 weeks (median differences: 1 and 2 points, respectively; both *P* < 0.001). This advantage persisted at 2 months (*P* = 0.002) and remained marginally significant at 3 months (*P* = 0.055), with continued superiority in pain control at 6 months (*P* = 0.040). (Table 3).

**Table 3:** Comparison of postoperative VAS pain scores between the two groups at different time points [Median(Q1,Q3)]

VAS score	Control group	SSVPI group	Z value	*P* value
B.S	8.00(6.25,9.00)	7.00(7.00,9.00)	−0.176	0.860
A.S(1 week)	3.00(2.00,3.75)	2.00(1.00,3.00)	−3.730	<0.001
A.S(2 weeks)	3.00(2.00,3.00)	1.00(1.00,2.00)	−4.918	<0.001
A.S(1 month)	2.00(1.00,2.00)	1.50(1.00,2.00)	−0.832	0.405
A.S(2 months)	3.00(1.00,5.00)	2.00(1.00,2.00)	−3.098	0.002
A.S(3 months)	2.00(2.00,3.75)	2.00(1.00,2.00)	−1.916	0.055
A.S(6 months)	3.00(2.00,4.00)	2.00(1.00,3.00)	−2.051	0.040
A.S(12 months)	5.00(4.00,5.00)	4.00(4.00,5.00)	−0.663	0.507

B.S: Before surgery

A.S: After surgery

### Functional status

#### KPS scores

No significant intergroup difference were observed in the preoperative KPS scores (*P* = 0.475), demonstrating comparability of the two groups. SSVPI group showed superior functional recovery: the 2-week KPS score was 80 compared with 70 for the control group (*P* = 0.006). (Table 4).

**Table 4:** Comparison of postoperative KPS scores between two groups at different follow-up time points [Median(Q1,Q3)]

KPS score	Control group	SSVPI group	Z value	*P* value
B.S	40.00(40.00,50.00)	40.00(32.50,50.00)	−0.714	0.475
A.S(1 week)	70.00(60.00,80.00)	80.00(62.50,90.00)	−1.799	0.072
A.S(2 weeks)	70.00(60.00,80.00)	80.00(70.00,90.00)	−2.751	0.006
A.S(1 month)	80.00(70.00,90.00)	80.00(80.00,90.00)	−0.641	0.522
A.S(2 months)	65.00(50.00,80.00)	75.00(60.00,80.00)	−1.489	0.137
A.S(3 months)	80.00(80.00,90.00)	80.00(70.00,90.00)	−1.189	0.235
A.S(6 months)	80.00(70.00,90.00)	80.00(60.00,90.00)	−1.173	0.241
A.S(12 months)	60.00(50.00,60.00)	60.00(50.00,60.00)	−1.205	0.228

B.S: Before surgery

A.S: After surgery

ECOG scores

There were no significant intergroup difference in pretreatment ECOG scores. At two weeks postoperatively, the SSVPI group exhibited better neurological recovery compared to the control group. The intergroup comparison showed a highly significant difference (Z = −4.189, *P* < 0.001) (Table 5).

**Table 5:** Comparison of ECOG performance status between two groups before and after therapy [Median(Q1,Q3)]

ECOG score	Control group	SSVPI group	Z value	*P* value
B.S	3.00(3.00,3.00)	3.00(2.25,3.00)	−0.110	0.912
A.S(2 weeks)	3.00(3.00,3.00)	2.00(2.00,3.00)	−4.189	<0.001

B.S: Before surgery

A.S: After surgery

#### WAS scores

There were no significant intergroup differences in preoperative WAS scores for both groups (Z=−0.288, *P* = 0.773). One week post-intervention, the control group showed significantly worse walking ability (median score: 4 (3–4), while the SSVPI group demonstrated improvement (median score: 1 (0.25–2.75). Intergroup comparisons revealed a marked difference (Z = −6.216, *P* < 0.001) (Table 6). Note: In this study, higher scores reflect better muscle strength.

**Table 6:** Comparison of WAS scores between two groups before and after therapy [Median(Q1,Q3)]

WAS score	Control group	SSVPI group	Z value	*P* value
B.S	0(0,2.00)	0(0,2.00)	−0.288	0.773
A.S(1 week)	1.00(0.25,2.75)	4.00(3.00,4.00)	−6.216	<0.001

B.S: Before surgery

A.S: After surgery

Lovett muscle strength grading

Preoperatively, the commonest grades of the control group were Grade 2 (29.2%) and Grade 4 (45.8%), whereas for the SSVPI group it was Grade 3 (45.8%), with the difference between the two groups being statistically significant (Z = −2.419, *P* = 0.016). Postoperative Outcomes (1-week follow-up): At one-week postoperatively the SSVPI group demonstrated superior improvement, with 77.1% achieving Grade 4 strength, and 1 patient recovered to normal Grade 5 strength. In the control group, only 39.6% of the patients had Grade 4 strength. The intergroup difference was significant (Z = −4.435, *P* < 0.001) (Table 7).

**Table 7:** Intergroup comparison of Lovett grades at baseline and one week after treatment [n(%)]

Lovett grade	Control group	SSVPI group	Z value	*P* value
B.S				
0	3(6.3)	5(10.4)	−2.419	0.016
1	5(10.4)	9(18.8)		
2	14(29.2)	9(18.8)		
3	4(8.3)	22(45.8)		
4	22(45.8)	3(6.3)		
A.S(1 week)				
0	1(2.1)	0	−4.435	<0.001
1	3(6.3)	0		
2	10(20.8)	0		
3	15(31.3)	10(20.8)		
4	19(39.6)	37(77.1)		
5	0	1(2.1)		

B.S: Before surgery

A.S: After surgery

#### Health-Related quality of life (HRQoL)

At baseline there were no significant difference in the median HRQoL scores between the groups; control group EORTC QLQ-C30 score 55.50 vs. SSVPI group 57.00 (*P* = 0.800). Post-treatment, the SSVPI group demonstrated significantly better EORTC QLQ-C30 score improvement compared to the control group: 1-week follow-up: 76.00 vs. 65.50 (*P* = 0.001); 1-month follow-up: 81.00 vs. 65.50 (*P* < 0.001); and 3-month follow-up: 81.50 vs. 78.00 (*P* = 0.003) (Table 8).

**Table 8:** Comparison of EORTC QLQ-C30 scores between control and SSVPI groups across treatment Phases[Median(Q1,Q3)]

C30 score	Control group	SSVPI group	Z value	*P* value
B.S	55.50(43.50,68.00)	57.00(37.25,69.50)	− 0.253	0.800
A.S(1 week)	65.50(50.00,73.50)	76.00(63.25,81.00)	−3.353	0.001
A.S(1 month)	65.50(50.00,73.50)	81.00(74.50,83.00)	−6.019	0.000
A.S(3 months)	78.00(65.00,82.00)	81.50(78.00,83.00)	−2.922	0.003

B.S: Before surgery

A.S: After surgery

### Survival outcome analysis

#### Overall survival (OS)

**Fig. 4:**
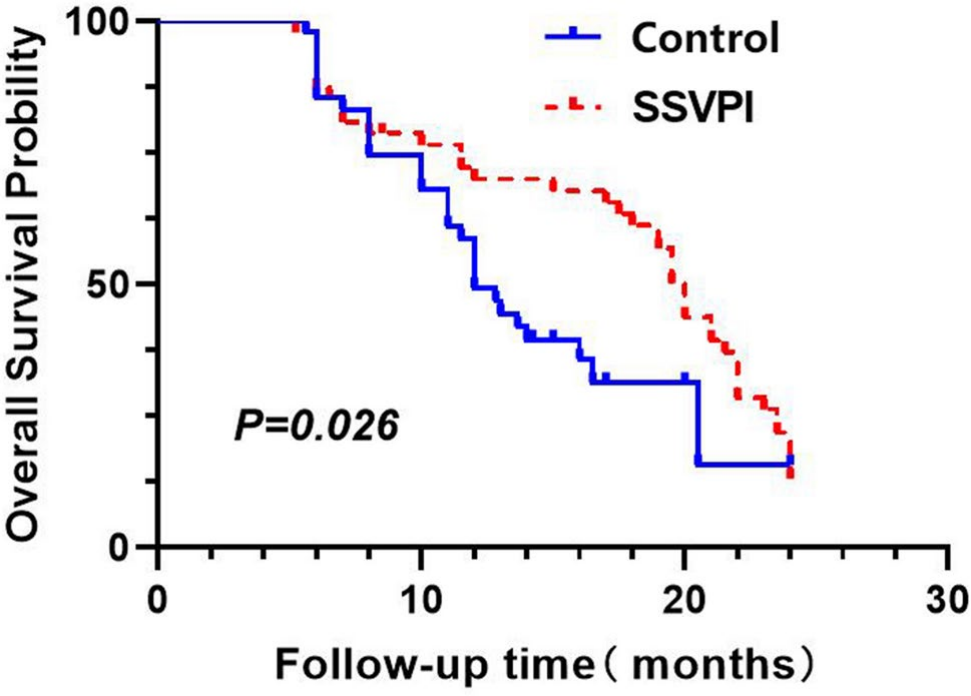
OS curves comparison between treatment groups

The median OS was 20 months (range: 5.23–24.23 months) for SSVPI group; and 12 months (range: 5.6–24 months) for the Control group. A statistically significant difference was observed between the two groups (*P* = 0.026) (Fig. 4).

#### Progression-free survival (PFS)

**Fig. 5:**
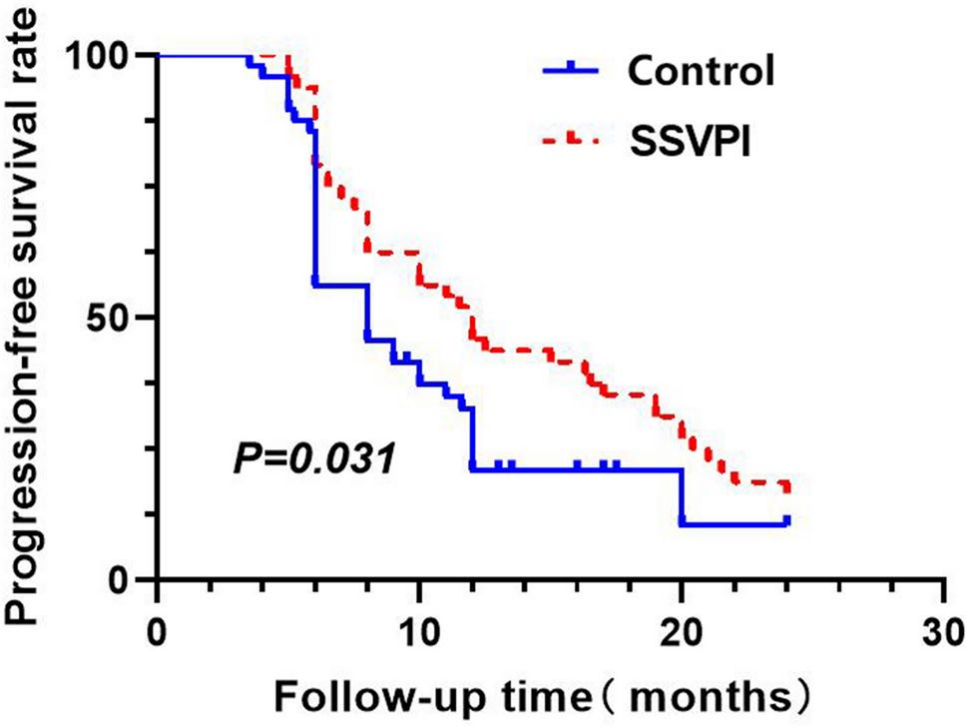
PFS curves comparison between treatment groups

The study SSVPI group had a median PFS of 12 months (range: 5–24 months), compared to a median PFS of 8 months (range: 3.5–24 months) for the control group. The intergroup difference was statistically significant (*P* = 0.031) (Fig. 5).

#### Local control (LC)

**Fig. 6:**
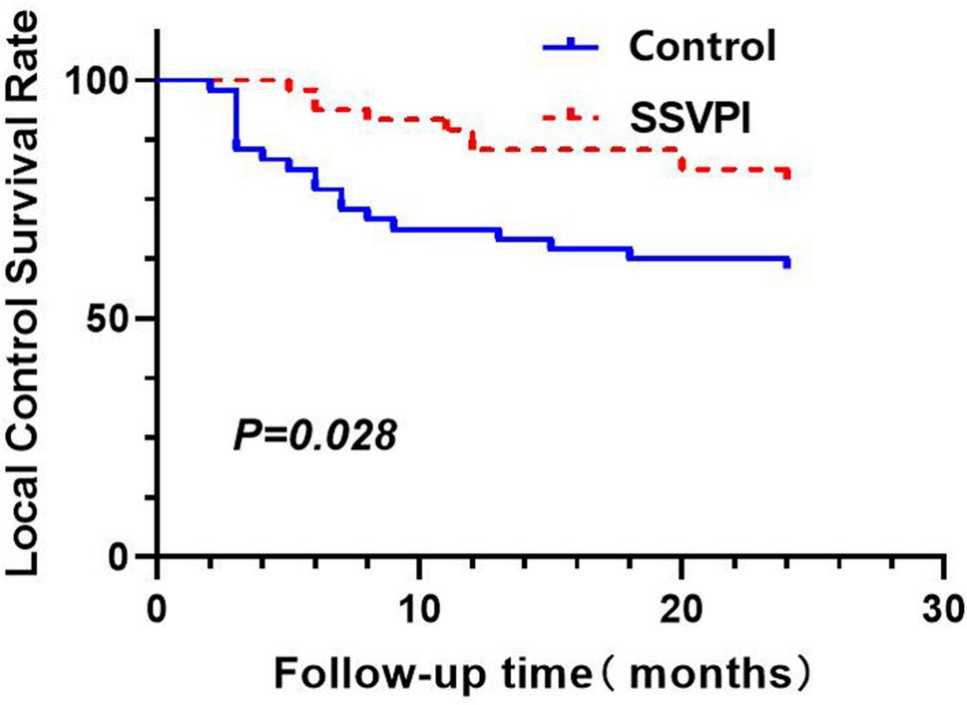
LC curves comparison between treatment groups

In the SSVPI group, the LC duration was 5–24 months, whereas for the control group it was 2–24 months. The difference between the two treatment groups was statistically significant (*P* = 0.028) (Fig. 6).

#### Complications

Complications in the SSVPI group included bone cement leakage (*n* = 5,10.42%), cerebrospinal fluid (CSF) leakage (*n* = 2, 4.17%), and Surgical site infection (*n* = 1,2.08%). No severe complications, including pneumothorax, pulmonary embolism, radiation myelitis, or secondary spinal cord compression, were observed.

Complications in the Control Group included cerebrospinal fluid leakage (*n* = 3, 6.25%), surgical site infection (*n* = 4, 8.33%), radiation-induced spinal cord injury with neurological deterioration (*n* = 2, 4.17%), radiation dermatitis (varying severity, *n* = 9, 18.75%), secondary spinal cord compression due to local progression (*n* = 3, 6.25%), and vertebral compression fractures from radiation-induced osteonecrosis (*n* = 8, 16.67%).

### Representative cases

#### Typical SSVPI case one

Patient Profile: Name: Dai XX, Gender: Male, Age: 52years, Preoperative Diagnosis:1)

L1 vertebral osteolytic destruction with epidural spinal cord compression (metastatic);2)

Incomplete paraplegia (ISNCSCI Grade C);3)Stage IV right lung adenocarcinoma with multiple systemic metastases.

Surgical Procedure: Under general anesthesia, the patient underwent: L1 Separation Surgery Combined with Intraoperative Vertebroplasty and ^125^I Seeds Implantation (SSVPI).

## Typical SSVPI case two

Patient Profile: Name: Xu XX, Gender: Female, Age: 50 years, Preoperative Diagnosis: 1) T10 vertebral osteolytic destruction with epidural spinal cord compression (metastatic); 2) Incomplete paraplegia (ISNCSCI Grade D); 3) Stage IV right lung adenocarcinoma with multiple systemic metastases.

Surgical Procedure: Under general anesthesia, the patient underwent: T10 Separation Surgery Combined with Intraoperative Vertebroplasty and ^125^I Seeds Implantation (SSVPI).

## Discussion

Spinal metastases represent the most common form of osseous dissemination in advanced malignancies [19], significantly compromising patients’ quality of life and prognosis. The management of spinal metastases aims to maintain or improve quality of life through multidisciplinary approaches, with key therapeutic elements including pain control, neurological preservation, and functional restoration [20, 21]. Conventional treatment paradigms relying on corticosteroids combined with external beam radiation therapy (EBRT) provide pain relief but demonstrate limited efficacy in restoring motor function.

Surgical intervention remains a cornerstone of the multidisciplinary management of spinal metastases. The surgical goals for spinal metastases include fracture prevention, spinal stabilization, functional recovery, and tumor debulking for symptom relief. Building on Building on the tumor-selective advantages of interstitial brachytherapy [18, 22], we developed PVPI for spinal metastases, capitalizing on the ideal properties of ^125^I (low energy, long half-life, cost-effectiveness) [23]. This combined approach effectively achieved pain relief, spinal stabilization, tumor control, and minimal complications [24–26]. Subsequent studies have confirmed that PVPI offers minimally invasive access, rapid pain relief, and low complication rates, establishing it as a viable alternative for managing spinal metastases [27, 28]. However, with spinal metastases, approximately 5–10% of patients [7, 8] develop irreversible paralysis due to spinal cord compression, significantly compromising their quality of life. Mechanical tumor compression directly damages the spinal cord tissue, causing local edema and venous congestion, while disrupting the blood-spinal cord barrier [29]. When compression persists beyond 72 h, it leads to secondary ischemic injury, in which microcirculatory disturbances can trigger irreversible infarction [30]. This pathological cascade constitutes the primary mechanism underlying neurological impairment in patients with MESCC. Conventional PVPI alone cannot address spinal cord compression.

Given that 85% of spinal metastases involve the anterior and middle vertebral columns, traditional posterior laminectomy not only fails to achieve adequate decompression but also compromises posterior spinal stability, increasing postoperative fracture risk by 42% [31]. While Tomita’s total en bloc spondylectomy (TES) demonstrates potential for improved local control [9], it has several limitations, including substantial intraoperative blood loss, technical complexity, and high complication rates (45.6% within 30 days with 19.8% requiring reoperation) [32]. A comparative study by Xu et al. [33] demonstrated that SS, originally proposed by Bilsky’s team, significantly reduced intraoperative bleeding and complication rates compared to radical procedures, such as TES. These findings were corroborated by Laufer et al. [13], whose research confirmed that SS maintains therapeutic efficacy while minimizing surgical complications. The landmark 2005 study by Patchell et al. [31] demonstrated superior ambulation preservation rates with surgery plus postoperative radiotherapy (84%) compared to radiotherapy alone (57%). This breakthrough approach leverages the immediate decompressive effect of surgery to compensate for radiotherapy’s delayed onset (typically 3–5 days), enabling timely intervention within the critical < 24-hour window for vascular spinal cord injury [34]. However, this approach has some limitations, including prolonged treatment intervals (2–3 weeks), vertebral fracture risk (26%), and radiation myelopathy (16%) [35–38]. To address issues, we combined SS with vertebroplasty and ^125^I seeds implantation for patients with MESCC.

This retrospective study systematically compared the efficacies of two surgical approaches for treating MESCC in patients with lung adenocarcinoma. The SSVPI group demonstrated superior outcomes, with 91.7% achieving ISNCSCI grade D at the 1-month follow-up (vs. 66.7% in the control group, *P* < 0.05) and maintaining this advantage for 6 months (91.7% vs. 62.5%, respectively). We propose that SSVPI achieves these outcomes through immediate decompression effect of separation surgery, with 78% of patients receiving intervention within the critical ≤ 72-hour window of neurological deficit onset, thereby mitigating hypoxia-induced apoptotic cascades [26]. Furthermore, vertebroplasty provides essential axial support, the concurrent intraoperative ^125^I seeds implantation enables immediate radiotherapy - a strategic advantage that prevents the exacerbation of neural edema associated with delayed radiotherapy administration.

The SSVPI group demonstrated significant early improvements in functional and quality-of-life metrics, including ECOG performance status, VAS pain scores, Lovett muscle strength recovery rate, and QLQ-C30 scores. Moreover, this approach conferred substantial survival benefits, with a median overall survival (OS) of 20 months—representing a 67% extension compared to the control group. We attribute the superior outcomes in the SSVPI group to three key mechanisms, including vertebroplasty with bone cement not only restores vertebral strength [39] and reconstructs spinal biomechanics, but also exerts immediate tumoricidal effects through cement monomer toxicity and polymerization-induced hyperthermia [27, 40–42], Concurrent ^125^I seeds implantation provides continuous low-dose radiation that effectively suppresses tumor growth [43], significantly prolonging local control. In addition this combined approach facilitates earlier resumption of systemic therapy, creating a synergistic treatment effect.

In this study, the SSVPI group demonstrated a distinct safety advantage. With cement leakage occurring in 10.42% of cases, lower than conventional PVP rates [44, 45]. This improvement is primarily attributed to the combination of continued use of beveled needles and optimized bone cement injection timing [46], along with the intraoperative direct visualization of cement injection, which enables earlier detection and management of cement leakage. While two CSF leaks occurred, consistent with reported complication rates for spinal surgery in the literature [47, 48]. All cases were successfully managed with conservative measures [49], including bed rest and lumbar drainage. whereas the control group experienced complications predominantly related to tumor progression-associated secondary spinal cord compression (3 cases, 6.25%) and radiation therapy-related adverse events (total incidence: 39.6%), including radiation dermatitis (18.75%), osteonecrosis (16.67%), and spinal cord injury (4.17%). Current evidence indicates that radiation-induced vertebral compression fractures typically occur 12–36 months post-radiotherapy [50], influenced by dosimetric [51–53], metabolic (osteoporosis/vitamin D deficiency) [54], and technical factors [55–57]. The SSVPI approach demonstrated certain prevention of radiation-associated fractures, attributable to ^125^I seeds’ precise dose distribution [58, 59], but its prolonged low-dose irradiation necessitates long-term monitoring for DNA damage accumulation. Notably, no secondary cord compression cases occurred, likely due to the cement-^125^I composite’s dual mechanism: biomechanical stabilization preserving spinal canal integrity combined with synergistic tumor control through cement cytotoxicity and sustained radiation effects.

The study has certain limitations, including its single-center design, limited sample size, and relatively short median follow-up period, which may affect the observation of long-term efficacy and delayed adverse events. Additionally, differences among participants in socioeconomic background, medical conditions, and lifestyle factors may also have some impact on the statistical power of the study results [60]. We will conduct multicenter randomized controlled trials with extended follow-up periods to evaluate long-term efficacy while optimizing particle dose distribution to reduce radiation exposure to surrounding tissues, thereby comprehensively assessing the clinical value of this technique.

## Conclusion

SSVPI is an optimized therapeutic strategy for lung adenocarcinoma patients with MESCC. It provides immediate spinal cord decompression and stabilization, concurrent intraoperative ^125^I seeds implantation for early and sustained radiotherapy, and avoids delayed postoperative radiation and complications of external beam therapy. This approach enhances local tumor control while preserving neurological function and improving quality of life.

## Data Availability

No datasets were generated or analysed during the current study.
